# Real-World Effectiveness of Boosting Against Omicron Hospitalization in Older Adults, Stratified by Frailty

**DOI:** 10.3390/vaccines13060565

**Published:** 2025-05-26

**Authors:** Liang En Wee, Enoch Xue Heng Loy, Jue Tao Lim, Wei Hao Kwok, Calvin Chiew, Christopher Lien, Barbara Helen Rosario, Ian Yi Onn Leong, Reshma Aziz Merchant, David Chien Boon Lye, Kelvin Bryan Tan

**Affiliations:** 1 National Centre for Infectious Diseases, Singapore 308442, Singapore; 2 Duke-NUS Medical School, National University of Singapore, Singapore 169857, Singapore; 3 Department of Infectious Diseases, Singapore General Hospital, Singapore 169608, Singapore; 4 Lee Kong Chian School of Medicine, Nanyang Technological University, Singapore 308232, Singapore; 5 Ministry of Health, Singapore 169854, Singapore; 6 Department of Geriatric Medicine, Changi General Hospital, Singapore 529889, Singapore; 7 Department of Geriatric Medicine, Tan Tock Seng Hospital, Singapore 308433, Singapore; 8 Division of Geriatric Medicine, Department of Medicine, National University Hospital, Singapore 119074, Singapore; mdcram@nus.edu.sg; 9 Yong Loo Lin School of Medicine, National University of Singapore, Singapore 117597, Singapore; 10 Department of Infectious Diseases, Tan Tock Seng Hospital, Singapore 308433, Singapore; 11 Saw Swee Hock School of Public Health, National University of Singapore, Singapore 117549, Singapore

**Keywords:** SARS-CoV-2, Omicron, vaccination, boosting, frailty, geriatrics

## Abstract

Background/Objectives: Older adults with frailty are at-risk of worse outcomes following respiratory-viral-infections such as COVID-19. Data on effectiveness of vaccination/boosting in frail older adults during Omicron is lacking. Methods: National healthcare-claims data and COVID-19 registries were utilized to enroll a cohort of older Singaporeans (≥60 years) as of 1 January 2022, divided into low/intermediate/high-risk for frailty; matching weights were utilized to adjust for sociodemographic differences/vaccination uptake at enrolment across frailty categories. Competing-risk-regression (Fine-Gray) taking death as a competing risk, with matching weights applied, was utilized to compare risks of COVID-19-related hospitalizations and severe COVID-19 across frailty levels (low/intermediate/high-risk), with estimates stratified by booster status. Individuals were followed up until study end-date (20 December 2023). Results: 874,160 older adults were included during Omicron-predominant transmission; ~10% had intermediate/high-frailty-risk. Risk of hospitalization/severe COVID-19 was elevated in those with intermediate/high-frailty-risk up to XBB/JN.1 transmission. Boosting was associated with decreased risk of COVID-19-related hospitalization across all frailty categories in infection-naïve individuals. However, in infection-naïve older adults with high-frailty-risk, while receipt of first boosters was associated with lower risk of COVID-19-hospitalization/severe COVID-19, additional booster doses did not reduce risk. In reinfected older adults, first boosters were still associated with lower hospitalization risk (adjusted-hazards-ratio, aHR = 0.55, 95% CI = 0.33–0.92) among the non-frail, but not in the intermediate/high-frailty-risk minority. Conclusions: First boosters were associated with reduced adverse COVID-19 outcomes across all frailty categories in infection-naïve older adults during Omicron. However, in the high-frailty minority, boosting did not additionally reduce risk in reinfected individuals with hybrid immunity, and beyond the first booster for infection-naïve individuals.

## 1. Introduction

Frailty reflects reduced physiological reserve from multi-systemic dysfunction and increased vulnerability arising from infective stressors, including COVID-19 [1]. In a systematic review, frailty was associated with poorer COVID-19-related outcomes [2]; however, most studies were conducted prior to emergence of the milder SARS-CoV-2 Omicron variant and rollout of vaccination [3], when mortality and morbidity attributed to COVID-19 substantially decreased [4]. Vaccination attenuates risk of adverse COVID-19 outcomes [5], but older adults have diminished vaccine responses due to immunosenescence [6,7], which may be exacerbated in frailty [8].

Given the Omicron variant’s more immune-evasive nature, booster doses are recommended in older adults to restore waning immunity. However, comparative studies of COVID-19 vaccine-effectiveness (VE) in frail versus non-frail older adults are limited to studies of primary vaccination conducted in the pre-Omicron era [9,10,11], which significantly limits generalizability pertaining to boosting, given the complex picture of hybrid immunity derived from successive booster doses and re-infections in the Omicron era. While protection against Omicron-related hospitalization plateaued after two vaccine doses among previously infected individuals in a large population-based cohort of older Canadians, results were not stratified by frailty [12]. As such, additional studies are required to guide vaccination recommendations for older adults living with frailty during COVID-19 endemicity, given that frailty potentially increases risk of adverse post-vaccination events [13]. We sought to evaluate the relationship between frailty and (a) adverse COVID-19 outcomes in the Omicron-predominant era; (b) protection conferred by successive boosting against adverse COVID-19 outcomes, stratified by frailty and by immunity from prior infection, in a highly boosted population-based cohort of older Singaporeans infected during successive waves driven by various Omicron subvariants, including XBB/JN.1 [14,15,16]. In Singapore, as part of the national vaccination program, BNT162b2 (Pfizer)/mRNA-1273 (Moderna) were originally approved as a two-dose primary series; ≥90% received mRNA vaccines [15]. Booster vaccinations (≥3 vaccine doses) were rolled out in September 2021 [15]. From December 2022 onwards, updated bivalent formulations fully replaced their monovalent predecessors [16]; monovalent XBB1.5 vaccines in turn replaced bivalent boosters in October 2023 [16]. We hypothesized that frailty remains associated with adverse COVID-19 outcomes even during milder Omicron infection, but that frailty may adversely impact the protection conferred by additional vaccine boosters during COVID-19 endemicity.

## 2. Materials and Methods

### 2.1. Study Population, Period and Design

Using a retrospective population-based cohort study design, national records of all confirmed SARS-CoV-2 infections/hospitalizations/COVID-19 vaccinations among older adults (citizens/permanent-residents) aged ≥ 60 years in Singapore, a Southeast Asian city-state, were linked with healthcare-claims records for the study period of 1 January 2022 to 20 December 2023 (Omicron-predominant transmission), based on national genomic surveillance (≥90% of sequenced cases). Individuals with missing sociodemographic data, who demised or were infected prior to study start date (enrolment date, 1 January 2022), were excluded. Individuals vaccinated with non-mRNA vaccines were also excluded, as they formed a non-representative minority (<5%) [15,16]. As the objective was to stratify COVID-19 outcomes by frailty, individuals who subsequently changed frailty category during the study period and could not be assigned to a single frailty category were additionally excluded. Omicron-predominant transmission was further subdivided into successive subvariant waves: BA.1/2 (6 January–30 March 2022) [14]; BA.4/5 (1 January–30 September 2022); XBB (18 October 2022–5 July 2023) [15]; and JN.1-predominant transmission (26 November–20 December 2023) [16]. COVID-19 outcomes and vaccination status were assessed using the national COVID-19 registry; national healthcare-claims data were utilized to provide information on comorbidities and classify frailty; and sociodemographic information was obtained from national databases maintained by the Ministry-of-Health (MOH). All data utilized for study analyses was anonymized.

### 2.2. Frailty

Frailty was defined using the Hospital-Frailty-Risk-Score (HFRS), which was calculated using International-Classification-of-Diseases, Tenth-Revision (ICD-10) codes obtained from the national healthcare-claims database (Mediclaims), at cohort enrolment (1 January 2022). Weights were assigned to ICD-10 codes using methodology described by Gilbert et al., [17] extended to include any healthcare encounter (hospitalizations/emergency-department/outpatient-visits) recorded in Mediclaims within the preceding 4 years. Although prior studies found that utilization of diagnostic codes beyond the preceding 2 years did not add significantly to HFRS’ predictive value [18], we utilized data from the preceding 4 years in order to mitigate potential differences in healthcare utilization during the COVID-19 pandemic by extending into the pre-pandemic period. The Mediclaims database encompasses all public/private healthcare providers in inpatient and outpatient settings; participation in the national government-administered medical-savings-scheme (Medisave) and national medical-insurance scheme (Medishield) is compulsory for Singaporeans. The population was stratified into low (HFRS < 5), intermediate (HFRS 5–15), and high (HFRS > 15) frailty risk [19,20]; the minority without a healthcare encounter in the preceding 4 years were classified as low frailty risk [20]. HFRS was utilized because of prior validation in the local setting, in the context of hospitalized older Singaporeans with community-acquired-pneumonia [19]; additionally, the nature of the HFRS as a frailty metric based on administrative healthcare-claims data meant that comprehensive healthcare-claims records with national-level coverage could be utilized to assess frailty on a population-wide level. As an alternative measure of frailty that involved direct patient assessment, the Clinical-Frailty-Scale (CFS) [21], comprising a 9-point scale (very fit-to-terminally ill) was measured by trained non-physician raters in a subset of the population who participated in a nationwide survey of community-dwelling adults aged ≥ 60 years, organized by our national Silver-Generation-Office (SGO) 2-years pre-pandemic (2019). CFS scores < 6 were classified as low risk of frailty; CFS scores of 6 were classified as intermediate risk of frailty; and CFS scores of 7–8 were considered at high risk of frailty. Individuals with CFS = 9 were dropped as they were approaching end-of-life. The CFS is Singapore’s nationally agreed frailty community screening tool [20].

### 2.3. COVID-19 Outcomes

Adverse COVID-19 outcomes were defined as COVID-19-related hospitalizations/severe COVID-19 occurring within the study period (1 January 2022–20 December 2023), recorded in the national COVID-19 registry. COVID-19-related hospitalization was defined as all-cause hospitalizations occurring within 3 days before or 14 days after a positive COVID-19 test; severe COVID-19 was defined as oxygen supplementation/intensive-care-unit (ICU)-admission/death during a COVID-19 hospitalization. During the study period, subsidized SARS-CoV-2 testing (polymerase-chain-reaction [PCR]/rapid-antigen-test [RAT]) was widely available across all healthcare providers (hospitals, public primary-care-clinics (polyclinics) and Public-Health-Preparedness-Clinics (PHPCs), a nationwide network of ≥1000 private general-practitioner clinics activated during pandemics [22]. Notification of all test-positive cases, COVID-19-related hospitalizations, and severe COVID-19 cases to MOH was mandatory throughout, with information recorded in the national COVID-19 registry [22]. During Omicron, risk of adverse COVID-19 outcomes was assessed for first infections and reinfections. Reinfection was taken as a repeat positive test (PCR/RAT) ≥ 90 days after initial infection.

### 2.4. Covariates

Demographics (age/sex/ethnicity), vaccination status, comorbidities (based on ICD-10 codes from Mediclaims) and SES were extracted from national databases. All covariates were defined at the point-of-infection, unless otherwise specified. SES was classified by housing-type, with purchase eligibility for smaller-sized, more heavily subsidized flats linked to household income [23]. Vaccination status was defined by number of mRNA vaccination doses recorded in the National-Immunization-Registry, at baseline (for weighting) and as a time-varying covariate in subsequent regressions (for analysis of risks for COVID-19 hospitalization/severe COVID-19).

### 2.5. Statistical Analysis

Categorical data were reported as percentages. Sociodemographic characteristics between older adults by frailty status (low/intermediate/high-risk) were reported as descriptive statistics, with standardized-mean-differences (SMDs) reported at baseline; matching weights, an extension of inverse-probability-of-treatment-weighting (IPTW) that allows simultaneous comparison across more than two groups [24], was subsequently utilized to account for between-group differences at baseline, with a SMD < 0.1 taken as evidence of good balance between frailty levels post-weighting. The following variables were included in weighting: age, ethnicity, gender, SES, comorbidity burden, and vaccination status pre-Omicron; vaccination status pre-Omicron was included given the association between prior vaccination and subsequent receipt of boosters [23]. Competing-risk-regression using Fine-Gray models, taking death as a competing risk and with matching weights applied, was subsequently conducted to compare risks of COVID-19-related hospitalizations and severe COVID-19 across frailty levels (low/intermediate/high-risk, HFRS), for first SARS-CoV-2 infections during Omicron-predominant transmission and reinfections during Omicron; the proportional-hazards assumption was tested by inspecting graphical plots of the scaled Schoenfeld residuals, and sensitivity analyses with cause-specific hazards models were conducted. Individuals were followed up to the end of the study period (20 December 2023), occurrence of the outcome event (COVID-19 hospitalization/severe COVID-19), or death, whichever occurred first; individuals who did not experience the outcome event or death were administratively censored at the end of the study. Number of vaccination doses (as a time-varying covariate), last vaccination type (ancestral versus updated vaccines [bivalent/monovalent XBB1.5 doses]), and time-since-last-vaccination were adjusted for in regression models. To assess if gender/SES modified the impact of frailty on adverse COVID-19 outcomes, interaction terms between gender/SES and frailty were included in regression models. As an additional analysis, risk estimates for COVID-19 outcomes during Omicron were stratified by successive Omicron waves (BA1/2, BA4/5, XBB, JN.1).

The following sensitivity analyses were conducted: (1) as HFRS was initially calculated using Mediclaims data for any healthcare encounter, analyses were restricted to individuals with ≥2 preceding hospitalizations for which HFRS could be computed [18]. (2) COVID-19 outcomes were evaluated using an alternative grouping of CFS categories (low frailty: CFS 1–3; moderate frailty: CFS 4–5; severe frailty: CFS 6–8), for the subset of the population who participated in the national Silver-Generation-Office (SGO) survey and had frailty assessed using CFS pre-pandemic. Agreement between the various grouping schemes utilized to condense the original CFS categories [21] into 3 categories for comparison against HFRS was assessed using the weighted kappa statistic [20]. As data were derived from national databases maintained by the MOH, data were complete; there was no missing data. Data were analyzed using R (version 4.3.1); *p* < 0.05 was considered statistically significant.

## 3. Results

**Figure 1:**
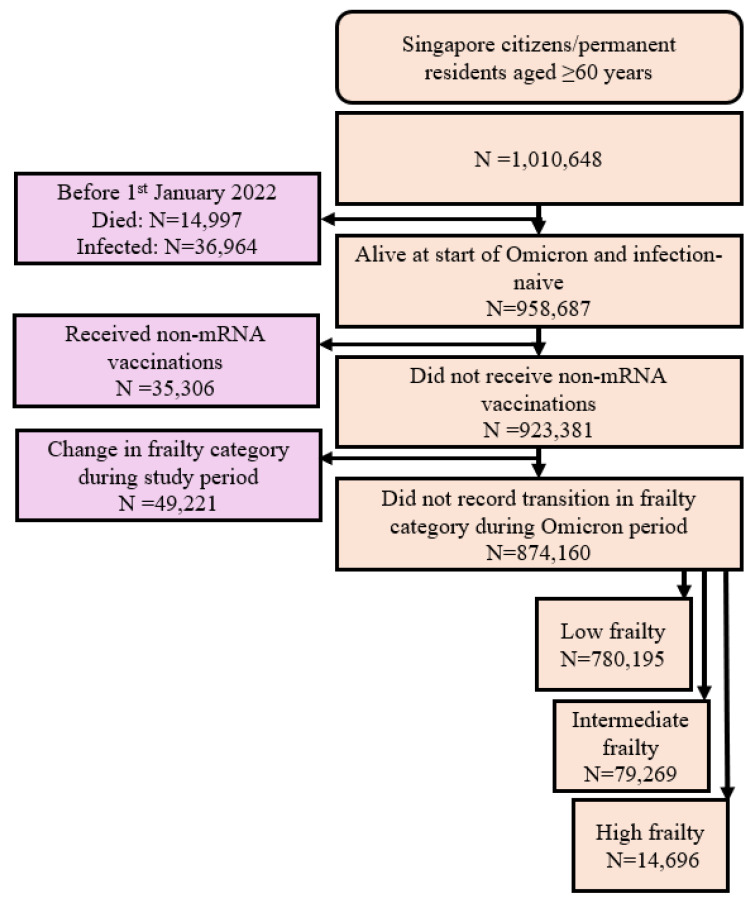
Cohort construction flowchart. Frailty risk was defined using the Hospital Frailty Risk Score (HFRS), stratified into the following categories: low (HFRS < 5), intermediate (HFRS 5–15), and high (HFRS > 15) risk of frailty.

Overall, after applying inclusion/exclusion criteria, during Omicron-predominant transmission, 874,160 older Singaporeans were included, with 89.3% (780,195/874,160), 9.1% (79,269/874,160) and 1.7% (14,696/874,160) classified as low, intermediate, and high-risk of frailty, respectively (Figure 1).

Post-weighting, SMDs were ≤0.1 between all frailty categories during Omicron-predominant transmission (Table 1). The median follow-up time was 366.4 days. A total of 319,074 first infections and 19,069 reinfections were recorded during Omicron, with 380 days (S.D = 157) on average elapsing between first infection and reinfection. Vaccination uptake was high overall, but lower among older adults with high frailty, versus those with low/intermediate-risk; a total of 578,850 booster doses were administered over the study period (Supplementary Table S1), with 66.8% (N = 386,617) comprising ancestral mRNA vaccine formulations; of those, 88.0% (N = 339,740) were BNT162b2, and 12.0% (N = 46,877) were mRNA-1273. The median time elapsed between receipt of a first booster dose and subsequent booster doses was 9.2 months. The proportion of various comorbidities, across frailty strata, is provided in Supplementary Table S2.

The adjusted-hazard-ratio(aHR) of COVID-19-related hospitalizations and severe COVID-19 in older adults during Omicron-predominant transmission, stratified by frailty, is shown in Table 2. During Omicron, a significantly elevated risk of COVID-19-related hospitalization and severe COVID-19 following both first infections and reinfections was observed in older adults with intermediate/high-risk of frailty, compared with those at low-risk of frailty (Table 2). Interaction terms between frailty and gender/SES were not significant (*p* > 0.05). Risk of COVID-19-related hospitalization and severe COVID-19 remained elevated in older adults with intermediate/high-risk of frailty, compared with those at low-risk of frailty, throughout successive Omicron waves, including XBB/JN.1 (Supplementary Table S3).

**Figure 2:**
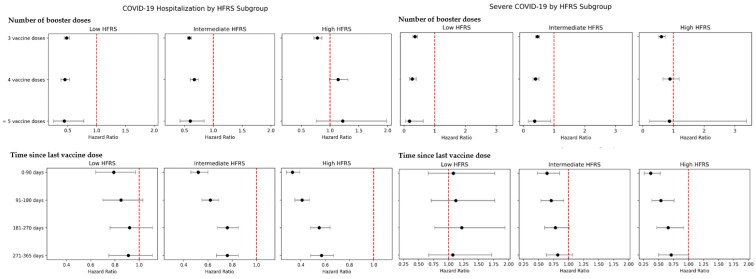
Risk of Omicron COVID-19 related-hospitalization and severe disease in infection-naïve older adult Singaporeans, by number of booster doses/time since last vaccination, stratified by frailty. Frailty risk was defined using the Hospital Frailty Risk Score (HFRS), stratified into the following categories: low (HFRS < 5), intermediate (HFRS 5-15) and high (HFRS > 15) risk of frailty. Hazards-ratio (HR) estimated utilizing competing risks regression (Fine-Gray), controlling for age, gender, ethnicity, socioeconomic status (housing type), comorbidities, vaccination status as of point-of-infection (number of doses, time elapsed from last vaccination dose, type of vaccine [ancestral mRNA vaccine versus updated bivalent/XBB1.5 vaccine formulation). For number of vaccine doses, full vaccination (two-dose regimen) was taken as the reference category; for time-since-last-dose, >365 days since last dose was taken as the reference category. HR < 1 indicates lower risk of COVID-19 hospitalization/severe COVID-19; dots indicate HRs and error bars indicate the 95% confidence-intervals.

Risks of Omicron COVID-19-related hospitalization and severe COVID-19 in infection-naive older Singaporeans, by number of vaccine doses and time elapsed since last vaccination, are reported in Figure 2/Supplementary Table S4, stratified by frailty. During Omicron-predominant transmission, in infection-naïve older adults at lowest risk of frailty (HFRS), risk of hospitalization (aHR = 0.48 [95% CI = 0.44–0.53]) and severe COVID-19 (aHR = 0.36 [95% CI = 0.29–0.44]) was significantly lower amongst those who had received ≥3 vaccine doses (boosted), versus 2-dose primary vaccination (Figure 2); with similar results when frailty was classified using CFS (Supplementary Table S4). In those at lowest risk of frailty (HFRS/CFS), lower risk of hospitalization and severe COVID-19 was also observed in those who received 4/≥5 vaccine doses (e.g. HFRS < 5, 4 vaccine doses: hospitalization, aHR = 0.45 [95% CI = 0.38–0.53], severe COVID-19: aHR = 0.27 [95% CI = 0.18–0.40]; ≥5 vaccine doses: hospitalization, aHR = 0.44 [95% CI = 0.25–0.78], severe COVID-19: aHR = 0.18 [95% CI = 0.05–0.63]) (Figure 2/Supplementary Table S4). In infection-naïve older adults at intermediate risk of frailty (HFRS/CFS), receipt of a 4th vaccine dose was similarly associated with lower risk of severe COVID-19 (aHR = 0.40 [95% CI = 0.32–0.51]), versus 2-dose primary vaccination. However, in infection-naïve older adults at highest risk of frailty (HFRS/CFS), while a third vaccine dose (first booster) was associated with lower risk of hospitalization (aHR = 0.78 [95% CI = 0.72–0.86]) and severe COVID-19 (aHR = 0.61 [95% CI = 0.52–0.73]) versus primary vaccination, no significant association was observed with additional booster doses beyond the first booster (e.g. HFRS ≥ 15, 4 vaccine doses: hospitalization, aHR = 1.14 [95% CI = 0.99–1.31]; severe COVID-19, aHR = 0.89 [95% CI = 0.66–1.19]) (Figure 2/Supplementary Table S4). In infection-naïve older adults with intermediate/high-risk of frailty, vaccination-induced protection against COVID-19 hospitalization lasted up to 365 days following the last vaccine dose (e.g.HFRS ≥ 15, 271–365 days post-vaccination: aHR = 0.57 [95% CI = 0.48–0.67]), while protection against severe COVID-19 lasted up to 180 days post-vaccination (e.g. HFRS ≥ 15, 91–180 days post-vaccination: aHR = 0.54 [95% CI = 0.39–0.76]) (Figure 2/Supplementary Table S4). Risk of COVID-19 hospitalization/severe COVID-19 did not significantly differ in older adults who received an updated vaccine formulation (versus ancestral vaccines), across all frailty categories (Supplementary Table S5).

**Figure 3:**
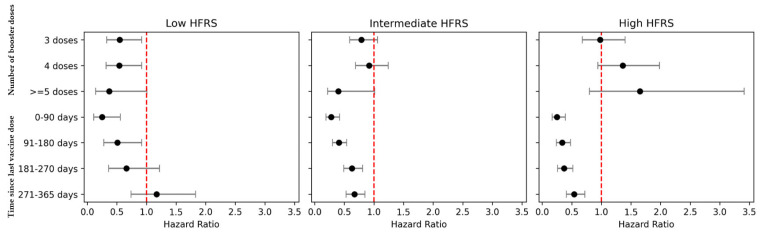
Risk of Omicron COVID-19 related-hospitalization in reinfected older adult Singaporeans, by number of booster doses/time since last vaccination, stratified by frailty.

Among individuals re-infected during Omicron, lower risk of COVID-19-related hospitalization was observed in reinfected older adults at low risk of frailty (HFRS < 5) who received 3 or 4 vaccine doses (3 vaccine doses: aHR = 0.55 [95% CI = 0.33–0.92]; 4 vaccine doses: aHR = 0.54 [95% CI = 0.32–0.92]), versus 2-dose primary vaccination (Figure 3/Supplementary Table S6). However, in reinfected older adults at intermediate/high risk of frailty, no significant difference in risk of COVID-19-related hospitalization was observed between boosted individuals and fully vaccinated individuals, although CIs were wide (e.g. HFRS ≥ 15, 4 vaccine doses: aHR = 1.36 [95% CI = 0.94–1.98]). In re-infected older adults with intermediate/high-risk of frailty, vaccination-induced protection against COVID-19 hospitalization lasted up to 365 days following the last vaccine dose (e.g. HFRS ≥ 15, 271–365 days post-vaccination: aHR = 0.54 [95% CI = 0.41–0.72]) (Figure 3/Supplementary Table S6). In sensitivity analyses, while the majority of individuals had a healthcare encounter in the preceding 4 years available for calculation of HFRS (Supplementary Table S7), restricting to individuals with ≥2 preceding hospitalizations (N = 134,863) did not significantly change risk estimates (Supplementary Tables S8 and S9). Usage of an alternative frailty measure (CFS) in the subset of the population with available data (N = 377,944, Supplementary Figure S1) yielded consistent results, though weak agreement (Cohen’s kappa < 0.40) was observed between CFS/HFRS categories (Supplementary Table S10). Use of an alternative grouping for CFS categories resulted in similar outcomes, with the risk of adverse COVID-19 outcomes significantly elevated in the highest frailty category by CFS (Supplementary Table S11). Similarly, across all categories of frailty (CFS), receipt of a first booster was associated with reduced risk of adverse COVID-19 outcomes during Omicron-predominant transmission; however, no significant reduction in COVID-19 hospitalization was observed among infection-naïve older adults in the highest frailty category (CFS) who received additional boosters (Supplementary Table S12).

## 4. Discussion

In a highly vaccinated/boosted population-based cohort of older Singaporeans, significantly elevated risk of adverse COVID-19 outcomes was observed in those with intermediate or high-risk of frailty, versus those at low-risk, throughout successive Omicron waves, including XBB/JN.1 subvariants. First boosters were associated with reduced adverse COVID-19 outcomes across all frailty categories in infection-naïve older adults during Omicron.

There is a paucity of evidence on COVID-19 vaccination efficacy and safety amongst frail older adults, given exclusion of this at-risk population from vaccine trials. Expert consensus is that frail older adults should be vaccinated except in situations of limited life expectancy [25], given the association between frailty and poorer COVID-19 outcomes in pre-Omicron studies [2,9,10,11]. However, effectiveness of vaccination decreases with frailty; in pre-Omicron studies, vaccine effectiveness of primary vaccination against COVID-19 hospitalization was much lower in frail older adults (36–63%), compared to non-frail older adults (65–77%) [10,11]. Our findings extend this to the context of Omicron-predominant transmission and booster vaccination; waning of protection with time elapsed from last vaccination and lack of significant protection afforded by additional boosters beyond the first booster were observed in infection-naïve frail older adults, versus individuals at low risk of frailty. These observations may be related to greater impact of immunosenescence in frail individuals, resulting in diminished titers of neutralizing antibodies and T-cell responses in frail versus non frail [7,26]. Additionally, frail older adults may have higher comorbidity burden; comorbidities such as inflammatory and cardiometabolic diseases were associated with reduced persistence of antibody responses to COVID-19 booster vaccination [27].

Waning immunity from vaccination in frail older adults during Omicron-predominant transmission carries significant implications, given potential for increased risk of frailty and other long-term sequelae post-COVID-19. Frailty remains common following COVID-19 hospitalization, with 65.5% of a cohort of COVID-19 survivors assessed as frail at 1-year follow-up [28]. Non-severe SARS-CoV-2 infection not resulting in hospitalization was still associated with a 66% increase in subsequent risk of frailty among older adults assessed as non-frail, prior to infection [29]. Frailty is also linked to elevated risk for long-term post-acute sequelae following COVID-19; in a US cohort, frailty was associated with a 41% increase in risk for long COVID-19 at a 6-month follow-up [30]. Given protection afforded by boosting against long COVID-19 during Omicron-predominant transmission [31,32,33,34], waning vaccine-effectiveness in frail older adults remains of concern.

Repeated administration of booster doses has been previously recommended as a strategy to restore waning immunity in older adults, given the more immune-evasive nature of the Omicron variant. In nursing home residents, boosting was required to develop detectable Omicron-specific neutralizing activity [35]. Our findings suggest that repeated boosting in the majority of older adults at intermediate-low risk of frailty remains a valid strategy during Omicron-predominant transmission, with lower risk of COVID-19-related hospitalization and severe COVID-19. However, in the subset of older adults living with frailty, reduction in risk of adverse COVID-19 outcomes was not observed beyond the first booster for infection-naïve individuals. In a longitudinal analysis of T-cell responses post-COVID-19 vaccination in frail nursing home residents, repeated boosters were unable to overcome statistically lower spike-specific T-cell responses in SARS-CoV-2–naive individuals [7]. In reinfected frail older adults with hybrid immunity, booster doses administered 6–9 months following the last vaccination dose did not additionally reduce risk of adverse COVID-19 outcomes, though no suggestion of negative vaccine efficacy due to immune imprinting was observed in our cohort [36]. Prior infection may stimulate underlying T-cell response that results in greater baseline protection; frail nursing home residents with prior infection had significantly higher T-cell response post-vaccination on longitudinal evaluation, versus infection-naïve subjects [7]. Breakthrough infection attributed to Omicron variants elicited higher specific immune responses than third dose (boosters) in healthy vaccinees [37]. While re-vaccination with updated vaccine formulations is still recommended given shifts in prevailing variants and superior protection against adverse COVID-19 outcomes [16,38,39,40,41,42,43,44], in frail older adults with higher risk of systemic adverse events post-vaccination/boosting [13,26,45], re-vaccination at longer intervals (e.g. yearly) can be considered, given that vaccination-induced protection against COVID-19 hospitalization lasted up to 365 days in both infection-naïve and re-infected frail individuals. Prioritization for COVID-19 therapeutics [46], and non-pharmacological preventive measures, such as masking and hand-hygiene [47,48,49,50,51], are still relevant during COVID-19 endemicity, among frail older adults where boosters afford more limited protection.

Our study has the following strengths: usage of a comprehensive nationwide vaccination/testing database during a period when subsidized diagnostic testing was widely available and strongly encouraged, minimizing misclassification of infection/vaccination status. National healthcare-claims data were used to compute HFRS. However, the following limitations exist. Although age, gender, ethnicity, SES and comorbidities were controlled for as covariates, unmeasured confounders may still introduce bias; for instance, although obesity is a risk factor for adverse COVID-19 outcomes [52,53], physical measurements (e.g. body-mass-index) were not available in national electronic-health-record data and hence could not be adjusted for. COVID-19 hospitalization was defined as all-cause hospitalizations occurring within 3 days before or 14 days after a positive COVID-19 test; while this definition might have included hospitalizations for other non-respiratory causes incidentally tested for COVID-19, during Omicron endemicity, SARS-CoV-2 testing was non-mandatory for hospitalized inpatients, and the top 10 ICD-10 diagnosis codes for notified COVID-19 hospitalizations (covering ≥65% of COVID-19 hospitalizations) were attributable to respiratory causes [54]. Use of HFRS, defined as using electronic-health-record data, to screen for frailty may not provide sufficient granularity to fully capture dynamic changes in functional status and is subject to coding accuracy, though assessment using CFS as a sensitivity analysis yielded comparable results. CFS was measured as a pre-pandemic baseline and not at the point of hospitalization, thus unmeasured changes in CFS pre-hospitalization could not be accounted for; though restrictions on home visitation during the pandemic meant that a more proximate assessment was unavailable. While there was weak concordance between HFRS/CFS in the classification of frailty, poor correlation between HFRS/CFS is known [55]; crucially, while various assessments of frailty may identify different population subsets as frail, this discordance did not significantly impact results. Frailty was associated with higher risk of adverse COVID-19 outcomes, and additional booster doses beyond the first booster did not significantly reduce the risk of COVID-19 hospitalization among infection-naïve adults in the highest frailty category, irrespective of the frailty metric used. Information on receipt of COVID-19 therapeutics (e.g. early nirmatrelvir/ritonavir), which may have potentially accounted for lower hospitalization risk in unvaccinated infection-naïve older adults who were prioritized for antivirals, was unavailable. However, <5% of eligible older Singaporeans received early nirmatrelvir/ritonavir [46].

## 5. Conclusions

Increased frailty correlated with increased hospitalization risk and severe COVID-19 in a cohort of older Singaporeans infected during successive Omicron waves. First boosters were associated with reduced adverse COVID-19 outcomes across all frailty categories in infection-naïve older adults during Omicron-predominant transmission. However, in the minority with high frailty, boosting did not additionally reduce risk of adverse COVID-19 outcomes in reinfected individuals with hybrid immunity and beyond the first booster for infection-naïve individuals.

## Figures and Tables

**Table 1:** Baseline characteristics of older Singaporean adults (N = 874,160) during Omicron-predominant transmission, stratified by frailty, with standardized-mean-differences before and after weighting. Frailty risk was defined using the Hospital Frailty Risk Score (HFRS), stratified into the following categories: low (HFRS < 5), intermediate (HFRS 5-15) and high (HFRS > 15) risk of frailty. Hazards-ratio (HR) estimated utilizing competing risks regression (Fine-Gray), controlling for age, gender, ethnicity, socioeconomic status (housing type), comorbidities, vaccination status as of point-of-infection (number of doses, time elapsed from last vaccination dose, type of vaccine [ancestral mRNA vaccine versus updated bivalent/XBB1.5 vaccine formulation). For number of vaccine doses, full vaccination (two-dose regimen) was taken as the reference category; for time-since-last-dose, >365 days since last dose was taken as the reference category. HR < 1 indicates lower risk of COVID-19 hospitalization; dots indicate HRs and error bars indicate the 95% confidence-intervals.

	Baseline, Before Weighting						Post-Weighting					
	Low Frailty ^a^, N(%)	Intermediate Frailty ^a^, N(%)	High Frailty ^a^, N(%)	SMD, Intermediate vs. Low Frailty	SMD, High vs. Low Frailty	SMD, High vs. Intermediate Frailty	Low ^a^, N(%)	Intermediate Frailty ^a^, N(%)	High Frailty ^a^, N(%)	SMD, Intermediate vs. Low R Frailty	SMD, High vs. Low f Frailty	SMD, High vs. Intermediate Frailty
**Age**												
60–69 years	477,130 (61.2%)	22,855 (28.8%)	2209 (15.0%)	0.69	1.08	0.34	114,226 (14.6%)	13,629 (17.2%)	2354 (16.0%)	0.07	0.04	0.03
70–79 years	226,332 (29.0%)	28,033 (35.4%)	3941 (26.8%)	0.14	0.05	0.19	214,366 (27.5%)	22,470 (28.3%)	4171 (28.4%)	0.02	0.02	0.00
≥80 years	76,733 (9.8%)	28,381 (35.8%)	8546 (58.2%)	0.65	1.19	0.46	451,603 (57.9%)	43,170 (54.5%)	8171 (55.6%)	0.07	0.05	0.02
**Ethnicity**												
Chinese	629,813 (80.7%)	62,161 (78.4%)	11,357 (77.3%)	0.06	0.08	0.03	611,531 (78.4%)	61,608 (77.7%)	11,399 (77.6%)	0.02	0.02	0.00
Indian	53,056 (6.8%)	6357 (8.0%)	1384 (9.4%)	0.05	0.10	0.05	70,410 (9.0%)	6869 (8.7%)	1358 (9.2%)	0.01	0.01	0.02
Malay	81,716 (10.5%)	9836 (12.4%)	1772 (12.1%)	0.06	0.05	0.01	89,258 (11.4%)	9787 (12.3%)	1749 (11.9%)	0.03	0.01	0.01
Others ^b^	15,610 (2.0%)	915 (1.2%)	183 (1.2%)	0.07	0.06	0.01	8996 (1.2%)	1006 (1.3%)	189 (1.3%)	0.01	0.01	0.00
**Gender**												
Female	412,040 (52.8%)	43,131 (54.4%)	8595 (58.5%)	0.03	0.11	0.08	449,928 (57.7%)	46,116 (58.2%)	8513 (57.9%)	0.01	0.01	0.01
Male	368,155 (47.2%)	36,138 (45.6%)	6101 (41.5%)	0.03	0.11	0.08	330,267 (42.3%)	33,153 (41.8%)	6183 (42.1%)	0.01	0.01	0.01
**Socioeconomic status (housing type) ^c^**												
Public, 1–2 room	52,033 (6.7%)	8228 (10.4%)	1884 (12.8%)	0.13	0.21	0.08	99,075 (12.7%)	9721 (12.3%)	1846 (12.6%)	0.01	0.00	0.01
Public, 3 room	145,828 (18.7%)	18,351 (23.2%)	3653 (24.9%)	0.11	0.15	0.04	199,443 (25.6%)	19,548 (24.7%)	3626 (24.7%)	0.02	0.02	0.00
Public, 4 room	239,768 (30.7%)	25,591 (32.3%)	4472 (30.4%)	0.03	0.01	0.04	237,066 (30.4%)	24,378 (30.8%)	4484 (30.5%)	0.01	0.00	0.01
Public, 5-room	254,797 (32.7%)	20,656 (26.1%)	3200 (21.8%)	0.15	0.25	0.10	173,046 (22.2%)	17,846 (22.5%)	3272 (22.3%)	0.01	0.00	0.01
Others	21,262 (2.7%)	1566 (2.0%)	643 (4.4%)	0.05	0.09	0.14	25,818 (3.3%)	2993 (3.8%)	601 (4.1%)	0.03	0.04	0.02
Private housing	66,507 (8.5%)	4877 (6.2%)	844 (5.7%)	0.09	0.11	0.02	45,747 (5.9%)	4782 (6.0%)	867 (5.9%)	0.01	0.00	0.01
**Comorbidity burden (Charlson Comorbidity Index, CCMI) ^d^**												
No comorbidities (CCMI = 0)	523,248 (67.1%)	19,035 (24.0%)	1381 (9.4%)	0.96	1.47	0.40	74,094 (9.5%)	7405 (9.3%)	1472 (10.0%)	0.01	0.02	0.02
Mild comorbidity burden (CCMI 1–2)	190,160 (24.4%)	26,978 (34.0%)	4875 (33.2%)	0.21	0.20	0.02	252,370 (32.3%)	29,827 (37.6%)	5171 (35.2%)	0.11	0.06	0.05
Moderate comorbidity burden (CCMI 3–4)	48,709 (6.2%)	19,275 (24.3%)	3998 (27.2%)	0.52	0.59	0.07	222,503 (28.5%)	21,663 (27.3%)	4083 (27.8%)	0.03	0.02	0.01
Severe comorbidity burden (CCMI ≥5)	18,078 (2.3%)	13,981 (17.6%)	4442 (30.2%)	0.53	0.82	0.30	231,228 (29.6%)	20,373 (25.7%)	3970 (27.0%)	0.09	0.06	0.03
**Vaccination status pre-Omicron ^e^**												
Unvaccinated/partially vaccinated	68,140 (8.7%)	3706 (4.7%)	1421 (9.7%)	0.16	0.03	0.19	61,548 (7.9%)	8368 (10.6%)	1358 (9.2%)	0.09	0.05	0.04
Fully vaccinated	126,919 (16.3%)	24,327 (30.7%)	6448 (43.9%)	0.35	0.63	0.28	339,195 (43.5%)	32,299 (40.7%)	6157 (41.9%)	0.06	0.03	0.02
Boosted	585,136 (75.0%)	51,235 (64.6%)	6826 (46.4%)	0.23	0.61	0.37	379,601 (48.6%)	38,598 (48.7%)	7180 (48.9%)	0.00	0.00	0.00

Data are n or n (%). SMD, standardized-mean-difference. ^a^ Frailty risk was defined using the Hospital Frailty Risk Score (HFRS), stratified into the following categories: low (HFRS < 5), intermediate (HFRS 5–15) and high (HFRS > 15) risk of frailty. ^b^ Includes individuals of other ethnicities or mixed ethnicities. ^c^ Housing type was used as an indicator of socioeconomic status. ^d^ Comorbidity burden was defined using the Charlson Comorbidity Index (CCMI), which consists of the following comorbidities: myocardial infarction, chronic heart failure, peripheral vascular disease, cerebrovascular accident, dementia, chronic obstructive pulmonary disease, connective tissue disease, peptic ulcer disease, diabetes mellitus, hemiplegia, liver disease, moderate to severe renal impairment, solid tumor, leukemia, human immunodeficiency virus (HIV) infection with AIDS. ^e^ For weighting, vaccination status was taken as of the start of study period (i.e., start of Omicron-predominant period), in order to weight by the likelihood of receiving COVID-19 vaccination (as receipt of prior COVID-19 vaccination would be a predictor of subsequent boosting during the Omicron-predominant period).

**Table 2:** Hazards-ratio of COVID-19 related-hospitalization and severe disease in older adult Singaporeans, stratified by frailty risk, during Omicron-predominant transmission.

Frailty Risk	Person-Years	Number of COVID-19 Hospitalizations	COVID-19 Hospitalization, aHR (95% CI) ^a^	Number of Severe COVID-19 cases	Severe COVID-19, aHR (95% CI) ^a^
**Omicron wave, first infections (infection-naïve)**					
**Frailty, defined using Hospital Frailty Risk Score (HFRS) ^b^**					
Low risk of frailty, HFRS < 5	1,106,573	12,577	1.00 (ref)	2008	1.00 (ref)
Intermediate risk of frailty, HFRS 5–15	94,698	9010	**2.03 (1.94, 2.13)**	1955	**2.44 (2.19, 2.72)**
High risk of frailty, HFRS > 15	14,949	3665	**3.38 (3.21, 3.56)**	927	**4.33 (3.86, 4.85)**
**Frailty, defined using Clinical Frailty Scale (CFS) in subset of population with available data ^c^**					
Low risk of frailty, CFS 1–5	475,826	10,378	1.00 (ref)	1683	1.00 (ref)
Intermediate risk of frailty, CFS 6	40,720	817	**1.79 (1.66, 1.94)**	176	**2.07 (1.75, 2.45)**
High risk of frailty, CFS 7–8	6428	1107	**1.89 (1.76, 2.03)**	318	**2.68 (2.33, 3.09)**
**Omicron wave, reinfections ^d^**					
**Frailty, defined using Hospital Frailty Risk Score (HFRS) ^b^**					
Low risk of frailty, HFRS < 5	1,466,967	928	1.00 (ref)	92	1.00 (ref)
Intermediate risk of frailty, HFRS 5–15	133,322	831	**2.68 (2.26, 3.19)**	114	**2.86 (1.77, 4.62)**
High risk of frailty, HFRS > 15	23,280	414	**5.29 (4.40, 6.35)**	60	**5.39 (3.27, 8.88)**

aHR: adjusted hazards-ratio; ref: reference category. ^a^ Competing-risks-regression (Fine-Gray), taking death as a competing risk, with matching weights applied; the following variables were included in weighting: age, ethnicity, gender, socioeconomic status, comorbidity burden, and vaccination status pre-Omicron. Vaccine type (ancestral mRNA vaccine formulation versus updated vaccines [bivalent/monovalent XBB1.5 doses]), number of vaccine doses (at point of infection), and time-since-last-vaccination were included as covariates in the regression models. ^b^ Frailty risk was defined using the Hospital Frailty Risk Score (HFRS), stratified into the following categories: low (HFRS < 5), intermediate (HFRS 5–15) and high (HFRS > 15) risk of frailty. ^c^ Frailty risk was defined using the Clinical Frailty Scale (CFS), in subset of population who participated in a large-scale nationwide survey of community-dwelling elderly, organized by the national Silver Generation Office (SGO) pre-pandemic (2019). The CFS is rated on nine levels: Level 1– Very Fit; Level 2—Fit; Level 3—Managing Well; Level 4—Living with Very Mild Frailty; Level 5—Living with Mild Frailty; Level 6—Living with Moderate Frailty; Level 7—Living with Severe Frailty; Level 8—Living with Very Severe Frailty; Level 9—Terminally Ill. CFS was categorized as follows: CFS scores <6 were classified as non-frail/mildly frail and therefore at low risk of frailty; CFS scores of 6 were classified as moderate frailty and therefore at intermediate risk of frailty; and CFS scores of 7–8 were considered severely frail and therefore at high risk of frailty. Individuals with CFS = 9 were dropped as they were approaching end-of-life. ^d^ Reinfections were defined as a repeat positive test for COVID-19 ≥90 days after initial infection. Only results stratified by HFRS are presented for reinfections; after restricting to subset of individuals who participated in the SGO survey and had CFS available, the number of reinfections during Omicron was small (N = 9622) and insufficient for additional analysis.

## Data Availability

The databases with individual-level information used for this study are not publicly available due to personal data protection. Deidentified data can be made available for research, subject to approval by the Ministry of Health of Singapore. All inquiries should be sent to the corresponding author.

## Supplementary Materials

The following supporting information can be downloaded at: https://www.mdpi.com/article/10.3390/vaccines13060565/s1; Table S1: Vaccination uptake amongst older adult Singaporeans during Omicron-predominant period, stratified by frailty risk. Table S2: Prevalence of comorbidities amongst older Singaporean adults (N = 874,160) during Omicron-predominant transmission, stratified by frailty. Table S3: Risk of COVID-19 related-hospitalization and severe disease in older adult Singaporeans, stratified by frailty risk, during successive Omicron waves (BA1/2, BA4/5, XBB, JN.1) Table S4: Risk of Omicron COVID-19 related-hospitalization and severe disease in infection-naïve older adult Singaporeans, by number of booster doses/time since last vaccination, stratified by frailty. Table S5: Risk of COVID-19 related-hospitalization and severe disease in older adult Singaporeans, by type of vaccination (ancestral/updated), stratified by frailty risk, during Omicron-predominant transmission. Table S6: Risk of Omicron COVID-19 related-hospitalization in reinfected older adult Singaporeans, by number of booster doses/time since last vaccination, stratified by frailty. Table S7: Percentage of older adult Singaporeans with healthcare utilization prior to study period. Table S8: Risk of COVID-19 related-hospitalization and severe disease in older adult Singaporeans with ≥2 preceding hospitalizations, stratified by frailty risk, during Omicron-predominant transmission. Table S9: Risk of COVID-19 related-hospitalization and severe disease in older adult Singaporeans with ≥2 preceding hospitalizations, by number of vaccine doses and time elapsed since last vaccination, stratified by frailty risk, during Omicron-predominant transmission. Table S10: Two-way cross-tabulation of frailty measures (Clinical-Frailty-Score and Hospital-Frailty-Risk-Score) in subset of the population with available data. Table S11: Risk of COVID-19 related-hospitalization and severe disease in older adult Singaporeans, stratified by alternative grouping using Clinical-Frailty-Scale, during Omicron-predominant transmission. Table S12: Risk of COVID-19 related-hospitalization and severe disease in older adult Singaporeans, by number of vaccine doses, stratified by alternative grouping using Clinical-Frailty-Scale, during Omicron-predominant transmission. Figure S1: Cohort construction flowchart for subset of population with Clinical-Frailty-Scale data available.

## Abbreviations

The following abbreviations are used in this manuscript:

VE	Vaccine effectiveness
HFRS	Hospital Frailty Risk Score
ICD-10	International-Classification-of-Diseases, Tenth-Revision
CFS	Clinical Frailty Scale
