# Supplementary material for: Real-World Effectiveness of Boosting Against Omicron Hospitalization in Older Adults, Stratified by Frailty
Source: Vaccines (Basel). 2025 May 26;13(6):565. doi: 10.3390/vaccines13060565 (PMC12197443; doi:10.3390/vaccines13060565)
Supplement: Supplementary file 1 [file vaccines-13-00565-s001.zip › vaccines-3589208-supplementary.pdf]

Supplementary Material, Table of Contents

- Supplementary Table 1: Vaccination uptake amongst older adult Singaporeans during Omicron-predominant period, stratified by frailty risk (pg. 2)
- Supplementary Table 2: Prevalence of comorbidities amongst older Singaporean adults (N=874,160) during Omicron-predominant transmission, stratified by frailty (pg. 3)
- Supplementary Table 3: Risk of COVID-19 related-hospitalization and severe disease in older adult Singaporeans, stratified by frailty risk, during successive Omicron waves (BA1/2, BA4/5, XBB, JN.1) (pg. 4)
- Supplementary Table 4. Risk of Omicron COVID-19 related-hospitalization and severe disease in infection-naïve older adult Singaporeans, by number of booster doses/time since last vaccination, stratified by frailty (pg.5,6)
- Supplementary Table 5: Risk of COVID-19 related-hospitalization and severe disease in older adult Singaporeans, by type of vaccination (ancestral/updated), stratified by frailty risk, during Omicron-predominant transmission (pg.7)
- Supplementary Table 6. Risk of Omicron COVID-19 related-hospitalization in reinfected older adult Singaporeans, by number of booster doses/time since last vaccination, stratified by frailty (pg.8)
- Supplementary Table 7: Percentage of older adult Singaporeans with healthcare utilization prior to study period (pg. 9)
- Supplementary Table 8: Risk of COVID-19 related-hospitalization and severe disease in older adult Singaporeans with ≥2 preceding hospitalisations, stratified by frailty risk, during Omicron-predominant transmission (pg.10)
- Supplementary Table 9: Risk of COVID-19 related-hospitalization and severe disease in older adult Singaporeans with ≥2 preceding hospitalisations, by number of vaccine doses and time elapsed since last vaccination, stratified by frailty risk, during Omicron-predominant transmission (pg. 11)
- Supplementary Table 10: Two-way cross-tabulation of frailty measures (Clinical-Frailty-Score and Hospital-Frailty-Risk-Score) in subset of the population with available data (pg. 12)
- Supplementary Table 11: Risk of COVID-19 related-hospitalization and severe disease in older adult Singaporeans, stratified by alternative grouping using Clinical-Frailty-Scale, during Omicron-predominant transmission (pg. 13)
- Supplementary Table 12: Risk of COVID-19 related-hospitalization and severe disease in older adult Singaporeans, by number of vaccine doses, stratified by alternative grouping using Clinical-Frailty-Scale, during Omicron-predominant transmission (pg.14)
- Supplementary Figure 1: Cohort construction flowchart for subset of population with Clinical-Frailty-Scale data available (pg.15)

Supplementary Table 1: Vaccination uptake amongst older adult Singaporeans at beginning and end of Omicron-predominant period, stratified by frailty risk

	Start of Omicron-predominant transmission (1st Jan 2022) N=874,160				End of study period, Omicron-predominant transmission (20th Dec 2023) N=874,160			
	Low risk of frailty, older adults (aged≥60 years) [a]	Intermediate risk of frailty, older adults (aged≥60 years)[a]	High risk of frailty, older adults (aged≥60 years) [a]	Whole adult population (aged≥18 years)	Low risk of frailty, older adults (aged≥60 years) [a]	Intermediate risk of frailty, older adults (aged≥60 years) [a]	High risk of frailty, older adults (aged≥60 years) [a]	Whole adult population (aged≥18 years)
**Total number**	**N=780,195**	**N=79,269**	**N=14,696**	**N=3,402,917**	**N=780,195**	**N=79,269**	**N=14,696**	**N=3,402,917**
**Vaccination status**								
Unvaccinated	66,672 (8.5%)	2,966 (3.7%)	1,058 (7.2%)	248,951 (7.3%)	64,914 (8.3%)	2,278 (2.9%)	764 (5.2%)	239,097 (7.0%)
1 vaccine dose	1,468 (0.2%)	740 (0.9%)	363 (2.5%)	11,310 (0.3%)	650 (0.1%)	264 (0.3%)	135 (0.9%)	4,427 (0.1%)
2 vaccine doses	126,919 (16.3%)	24,327 (30.7%)	6,448 (43.9%)	1,503,608 (44.2%)	12,002 (1.5%)	3,854 (4.9%)	1,417 (9.6%)	91,586 (2.7%)
3 vaccine doses	585,117 (75.0%)	51,230 (64.6%)	6,825 (46.4%)	1,638,956 (48.2%)	315,850 (40.5%)	32,319 (40.8%)	5,699 (38.8%)	1,938,022 (57.0%)
4 vaccine doses	19 (0.0%)	5 (0.0%)	1 (0.0%)	88 (0.0%)	291,081 (37.3%)	31,365 (39.6%)	5,437 (36.4%)	965,364 (28.4%)
≥5 vaccine doses	0 (0.0%)	1 (0.0%)	1 (0.0%)	4 (0.0%)	95,698 (12.3%)	9,189 (11.6%)	1,334 (9.1%)	164,421 (4.8%)
Fully-vaccinated (≥2 vaccine doses)	712,055 (91.3%)	75,563 (95.3%)	13,275 (90.3%)	3,142,656 (92.4%)	714,631 (91.6%)	76,727 (96.8%)	13,887 (94.5%)	3,159,393 (92.8%)
Boosted (≥3 vaccine doses)	585,136 (75.0%)	51,236 (64.6%)	6,827 (46.5%)	1,639,048 (48.2%)	702,629 (90.1%)	72,873 (91.9%)	12,470 (84.9%)	3,067,807 (90.2%)
Doubly boosted (≥4 vaccine doses)	19 (0.0%)	6 (0.0%)	2 (0.0%)	92 (0.0%)	386,779 (49.6%)	40,554 (51.2%)	6,771 (46.1%)	1,129,785 (33.2%)
**Vaccination type**								
Received updated vaccine (bivalent/XBB1.5)	0 (0.0%)	0 (0.0%)	0 (0.0%)	47 (0.0%)	174,141 (22.3%)	15,578 (19.7%)	2,514 (17.1%)	718,495 (21.1%)

[a] Frailty risk was defined using the Hospital Frailty Risk Score (HFRS), stratified into the following categories: low (HFRS<5), intermediate (HFRS 5-15) and high (HFRS>15) risk of frailty.

Supplementary Table 2: Prevalence of comorbidities amongst older Singaporean adults (N=874,160) during Omicron-predominant transmission, stratified by frailty

Specific comorbidities	Low risk of frailty[a], N(%)	Intermediate risk of frailty[a], N(%)	High risk of frailty[a], N(%)
Ischemic heart disease	63,251 (8.1%)	16,329 (20.6%)	3,688 (25.1%)
Heart failure	5,637 (0.7%)	4,667 (5.9%)	1,692 (11.5%)
Cerebrovascular disease	26,395 (3.4%)	16,398 (20.7%)	4,911 (33.4%)
Chronic-obstructive-pulmonary disease	26,014 (3.3%)	7,361 (9.3%)	1,785 (12.1%)
Chronic renal impairment	30,749 (3.9%)	17,663 (22.3%)	4,004 (27.2%)
Dementia	4,128 (0.5%)	7,444 (9.4%)	5,358 (36.5%)
Malignancy	45,947 (5.9%)	9,726 (12.3%)	1,618 (11.0%)

Data are n or n (%). SMD, standardised-mean-difference.

[a]Frailty risk was defined using the Hospital Frailty Risk Score (HFRS), stratified into the following categories: low (HFRS<5), intermediate (HFRS 5-15) and high (HFRS>15) risk of frailty.

Supplementary Table 3: Risk of COVID-19 related-hospitalization and severe disease in older adult Singaporeans, stratified by frailty risk, during successive Omicron waves (BA1/2, BA4/5, XBB, JN.1)

	Number of events	COVID-19 hospitalization, aHR (95%CI)[a]	Number of events	Severe COVID-19 disease, aHR (95%CI)[a]
**Omicron BA.1/2[b]**				
Low risk of frailty[c]	4267	1.00 (ref)	749	1.00 (ref)
Intermediate risk of frailty[c]	3479	2.10 (1.95, 2.27)	816	2.51 (2.12, 2.96)
High risk of frailty[c]	1480	3.74 (3.45, 4.06)	433	5.16 (4.33, 6.14)
**Omicron BA.4/5[b]**				
Low risk of frailty[c]	3165	1.00 (ref)	487	1.00 (ref)
Intermediate risk of frailty[c]	2527	2.23 (2.03, 2.45)	538	2.50 (2.03, 3.10)
High risk of frailty[c]	1092	4.40 (3.98, 4.86)	257	5.03 (4.02, 6.29)
**Omicron XBB subvariants[b]**				
Low risk of frailty[c]	2391	1.00 (ref)	438	1.00 (ref)
Intermediate risk of frailty[c]	1884	2.16 (1.95, 2.40)	423	2.47 (1.96, 3.11)
High risk of frailty[c]	791	4.24 (3.79, 4.75)	177	4.51 (3.51, 5.80)
**Omicron JN.1 subvariants[b]**				
Low risk of frailty[c]	464	1.00 (ref)	23	1.00 (ref)
Intermediate risk of frailty[c]	440	2.28 (1.84, 2.83)	23	3.25 (1.39, 7.56)
High risk of frailty[c]	207	5.14 (4.07, 6.49)	7	3.97 (1.48, 10.61)

aHR: adjusted hazards-ratio; ref: reference category

[a] Competing-risks-regression (Fine-Gray), taking death as a competing risk, with matching weights applied; the following variables were included in weighting: age, ethnicity, gender, socioeconomic status, comorbidity burden, and vaccination status pre-Omicron. Vaccine type (ancestral mRNA vaccine formulation versus updated vaccines [bivalent/monovalent XBB1.5 doses]), number of vaccine doses, and time-since-last-vaccination were included as covariates in the regression models.

[b] Variant was assigned based on predominant circulating SARS-CoV-2 variant (≥90% of sequenced cases on national genomic surveillance). Omicron BA.1/2-predominant transmission was defined as infection occurring from 6th Jan 2022 to 30th Mar 2022; Omicron BA.4/5-predominant transmission was defined as infection occurring from 1st Jun 2022 to 30th Sep 2022; Omicron XBB-predominant transmission was defined as infection occurring from 18th Oct 2022 to 5th Jul 2023; and Omicron JN.1-predominant transmission was defined as infection occurring from 26th Nov 2023 to 20th Dec 2023. Number of hospitalisations (N=22,187) and severe COVID-19 cases (N=4,371) do not add up to the original number of hospitalisations and severe COVID-19 cases during the entirety of the Omicron period, as some cases were hospitalised during the transition between Omicron subvariants where a specific Omicron subvariant could not be definitively assigned.

[c] Frailty risk was defined using the Hospital Frailty Risk Score (HFRS), stratified into the following categories: low (HFRS<5), intermediate (HFRS 5-15) and high (HFRS>15) risk of frailty.

Supplementary Table 4. Risk of Omicron COVID-19 related-hospitalization and severe disease in infection-naïve older adult Singaporeans, by number of booster doses/time since last vaccination, stratified by frailty

Omicron wave, first infections	COVID-19 hospitalization						Severe COVID-19 disease					
Number of vaccine doses, at point of infection[d]	Low risk of frailty (HFRS<5)[a], N(%)		Intermediate risk of frailty (HFRS 5-15)[a], N(%)		High risk of frailty (HFRS>15)[a], N(%)		Low risk of frailty (HFRS<5)[a], N(%)		Intermediate risk of frailty (HFRS 5-15)[a], N(%)		High risk of frailty (HFRS>15)[a], N(%)	
	Events, per 1000 person-years	aHR (95%CI)[b]	Events, per 1000 person-years	aHR (95%CI)[b]	Events, per 1000 person-years	aHR (95%CI)[b]	Events, per 1000 person-years	aHR (95%CI)[b]	Events, per 1000 person-years	aHR (95%CI)[b]	Events, per 1000 person-years	aHR (95%CI)[b]
2 vaccine doses	50	1.00 (ref)	253	1.00 (ref)	433	1.00 (ref)	11	1.00 (ref)	64	1.00 (ref)	130	1.00 (ref)
3 vaccine doses	13	0.48 (0.44, 0.53)	96	0.58 (0.55, 0.62)	245	0.78 (0.72, 0.86)	2	0.36 (0.29, 0.44)	19	0.46 (0.40, 0.53)	58	0.61 (0.52, 0.73)
4 vaccine doses	8	0.45 (0.38, 0.53)	54	0.67 (0.60, 0.74)	143	1.14 (0.99, 1.31)	1	0.27 (0.18, 0.40)	9	0.40 (0.32, 0.51)	30	0.89 (0.66, 1.19)
≥5 vaccine doses	5	0.44 (0.25, 0.78)	26	0.60 (0.42, 0.84)	76	1.22 (0.76, 1.98)	1	0.18 (0.05, 0.63)	3	0.37 (0.16, 0.89)	8	0.87 (0.22, 3.38)
Number of vaccine doses, at point of infection[d]	Low risk of frailty (CFS 1-5)[c], N(%)		Intermediate risk of frailty (CFS 6)[c], N(%)		High risk of frailty (CFS 7-8)[c], N(%)		Low risk of frailty (CFS 1-5)[c], N(%)		Intermediate risk of frailty (CFS 6)[c], N(%)		High risk of frailty (CFS 7-8)[c], N(%)	
	Events, per 1000 person-years	aHR (95%CI)[b]	Events, per 1000 person-years	aHR (95%CI)[b]	Events, per 1000 person-years	aHR (95%CI)[b]	Events, per 1000 person-years	aHR (95%CI)[b]	Events, per 1000 person-years	aHR (95%CI)[b]	Events, per 1000 person-years	aHR (95%CI)[b]
2 vaccine doses	91	1.00 (ref)	214	1.00 (ref)	248	1.00 (ref)	20	1.00 (ref)	51	1.00 (ref)	84	1.00 (ref)
3 vaccine doses	21	0.54 (0.48, 0.59)	131	0.90 (0.74, 0.99)	143	0.83 (0.70, 0.98)	3	0.42 (0.34, 0.51)	29	0.72 (0.49, 0.98)	35	0.58 (0.43, 0.79)
4 vaccine doses	13	0.49 (0.42, 0.58)	77	1.01 (0.75, 1.36)	83	1.05 (0.80, 1.39)	2	0.34 (0.25, 0.46)	10	0.50 (0.26, 0.95)	18	0.71 (0.40, 1.25)
≥5 vaccine doses	7	0.32 (0.19, 0.53)	42	0.88 (0.33, 2.39)	75	1.58 (0.65, 3.81)	1	0.21 (0.07, 0.63)	5	0.74 (0.69, 1.54)	16	0.87 (0.16, 4.60)
Time elapsed since last vaccine dose[e]	Low risk of frailty (HFRS<5)[a], N(%)		Intermediate risk of frailty (HFRS 5-15)[a], N(%)		High risk of frailty (HFRS>15)[a], N(%)		Low risk of frailty (HFRS<5)[a], N(%)		Intermediate risk of frailty (HFRS 5-15)[a], N(%)		High risk of frailty (HFRS>15)[a], N(%)	
	Events, per 1000 person-years	aHR (95%CI)[b]	Events, per 1000 person-years	aHR (95%CI)[b]	Events, per 1000 person-years	aHR (95%CI)[b]	Events, per 1000 person-years	aHR (95%CI)[b]	Events, per 1000 person-years	aHR (95%CI)[b]	Events, per 1000 person-years	aHR (95%CI)[b]
0-90 days	13	0.79 (0.64, 0.97)	112	0.52 (0.46, 0.60)	275	0.33 (0.28, 0.39)	2	1.08 (0.66, 1.77)	26	0.64 (0.48, 0.85)	67	0.37 (0.26, 0.53)
91-180 days	17	0.85 (0.70, 1.03)	134	0.62 (0.55, 0.69)	322	0.41 (0.35, 0.47)	3	1.12 (0.71, 1.77)	30	0.71 (0.54, 0.92)	92	0.54 (0.39, 0.76)

181-270 days	12	0.92 (0.76, 1.11)	94	0.76 (0.68, 0.85)	253	0.55 (0.48, 0.64)	2	1.22 (0.77, 1.94)	20	0.78 (0.60, 1.01)	63	0.66 (0.47, 0.92)
271-365 days	11	0.91 (0.75, 1.11)	86	0.76 (0.67, 0.85)	190	0.57 (0.48, 0.67)	2	1.07 (0.67, 1.72)	18	0.82 (0.63, 1.07)	47	0.71 (0.50, 1.00)
> 365 days	6	1.00 (ref)	49	1.00 (ref)	134	1.00 (ref)	1	1.00 (ref)	8	1.00 (ref)	25	1.00 (ref)

aHR: adjusted hazards-ratio; ref: reference category

[a] Frailty risk was defined using the Hospital Frailty Risk Score (HFRS), stratified into the following categories: low (HFRS<5), intermediate (HFRS 5-15) and high (HFRS>15) risk of frailty.

[b] Competing risks regression (Fine-Gray), controlling for age, gender, ethnicity, socioeconomic status (housing type), comorbidities, vaccination status as of point-of-infection (number of doses, time elapsed from last vaccination dose, type of vaccine [ancestral mRNA vaccine versus updated bivalent/XBB1.5 vaccine formulation)

[c] Frailty risk was alternatively defined using the Clinical Frailty Scale (CFS), in subset of population who participated in a large-scale nationwide survey of community-dwelling elderly, organised by the national Silver Generation Office (SGO) pre-pandemic (2019). The CFS is rated on 9 levels: Level 1– Very Fit; Level 2 – Fit; Level 3 – Managing Well; Level 4 – Living with Very Mild Frailty; Level 5 – Living with Mild Frailty; Level 6 – Living with Moderate Frailty; Level 7 – Living with Severe Frailty; Level 8 – Living with Very Severe Frailty; Level 9 – Terminally Ill. CFS scores <6 were classified as non-frail/mildly frail and hence low risk of frailty; CFS scores of 6 were classified as moderate and therefore intermediate risk of frailty; and CFS scores of 7-8 were considered severely frail and therefore at high risk of frailty. Individuals with CFS=9 were dropped as they were approaching end-of-life.

[d] COVID-19 vaccination status was determined at the point of SARS-CoV-2 infection. Full vaccination was defined as having completed a primary 2-dose vaccine series (either BNT162b2 or mRNA-1273, at least 8 weeks apart). Fully-vaccinated individuals who received additional mRNA vaccine doses at least six to nine months after the second dose were considered boosted. Additional vaccine doses were recommended to be administered around 1 year (and no earlier than 5 months) after the last dose received. Full vaccination was used as the reference category; the category of unvaccinated/partially vaccinated individuals was not presented due to the small number of unvaccinated/partially vaccinated cases in our population.

[e] Vaccination was considered effective 7 days after receipt to allow time for immune response. Estimates were presented from regression in which frailty risk was stratified using the Hospital Frailty Risk Score (HFRS).

Supplementary Table 5: Risk of COVID-19 related-hospitalization and severe disease in older adult Singaporeans, by type of vaccination, stratified by frailty risk, during Omicron-predominant transmission

	**COVID-19 hospitalization**						**Severe COVID-19 disease**					
	**Low risk of frailty[a], N(%)**		**Intermediate risk of frailty[a], N(%)**		**High risk of frailty[a], N(%)**		**Low risk of frailty[a], N(%)**		**Intermediate risk of frailty[a], N(%)**		**High risk of frailty[a], N(%)**	
	**Events, per 1000 person-years**	**aHR (95%CI)[b]**	**Events, per 1000 person-years**	**aHR (95%CI)[b]**	**Events, per 1000 person-years**	**aHR (95%CI)[b]**	**Events, per 1000 person-years**	**aHR (95%CI)[b]**	**Events, per 1000 person-years**	**aHR (95%CI)[b]**	**Events, per 1000 person-years**	**aHR (95%CI)[b]**
**Omicron wave, first infections (infection-naïve)**												
**Type of vaccine dose[c]**												
Ancestral monovalent	13	1.00 (ref)	96	1.00 (ref)	247	1.00 (ref)	2	1.00 (ref)	20	1.00 (ref)	61	1.00 (ref)
Updated vaccine	4	1.04 (0.65, 1.68)	25	1.07 (0.81, 1.41)	72	1.19 (0.83, 1.72)	0	1.59 (0.56, 4.55)	4	1.13 (0.58, 2.21)	8	0.64 (0.23, 1.78)

aHR: adjusted hazards-ratio; ref: reference category

[a] Frailty risk was defined using the Hospital Frailty Risk Score (HFRS), stratified into the following categories: low (HFRS<5), intermediate (HFRS 5-15) and high (HFRS>15) risk of frailty.

[b] Competing risks regression (Fine-Gray), controlling for age, gender, ethnicity, socioeconomic status (housing type), comorbidities, vaccination status (number of doses, time elapsed from last vaccination dose, type of vaccine [ancestral mRNA vaccine versus updated bivalent/XBB1.5 vaccine formulation), risk of frailty during Omicron-predominant transmission.

[c] Type of vaccine was defined as the type of mRNA vaccine received most proximate to SARS-CoV-2 infection, and classified as ancestral monovalent mRNA vaccine (BNT162b2/mRNA-1273) versus updated vaccine formulations (bivalent/monovalent XBB1.5 vaccine formulation). Vaccination was considered effective 7 days after receipt to allow time for immune response. As only a small proportion of the population were re-infected and a minority of those who were reinfected had received an updated vaccine dose, numbers of hospitalization and severe-COVID-19 events were insufficient to allow for comparison by type of vaccine dose in re-infected individuals.

Supplementary Table 6. Risk of Omicron COVID-19 related-hospitalization in reinfected older adult Singaporeans, by number of booster doses/time since last vaccination, stratified by frailty

Omicron wave, reinfections	COVID-19 hospitalization					
	Low risk of frailty (HFRS<5)[a], N(%)		Intermediate risk of frailty (HFRS 5-15)[a], N(%)		High risk of frailty (HFRS>15)[a], N(%)	
	Events, per 1000 person-years	aHR (95%CI)[b]	Events, per 1000 person-years	aHR (95%CI)[b]	Events, per 1000 person-years	aHR (95%CI)[b]
**Number of vaccine doses, at point of infection[c]**						
2 vaccine doses	1	1.00 (ref)	8	1.00 (ref)	15	1.00 (ref)
3 vaccine doses	1	0.55 (0.33, 0.92)	5	0.79 (0.59, 1.06)	14	0.98 (0.68, 1.40)
4 vaccine doses	1	0.54 (0.32, 0.92)	8	0.92 (0.69, 1.24)	23	1.36 (0.94, 1.98)
≥5 vaccine doses	1	0.37 (0.14, 1.01)	5	0.40 (0.22, 1.01)	30	1.65 (0.80, 3.41)
**Time elapsed since last vaccine dose**						
0-90 days[d]	0	0.25 (0.11, 0.56)	2	0.28 (0.19, 0.42)	6	0.25 (0.17, 0.39)
91-180 days	0	0.51 (0.28, 0.92)	3	0.41 (0.30, 0.54)	8	0.34 (0.24, 0.48)
181-270 days	0	0.66 (0.36, 1.22)	4	0.63 (0.49, 0.81)	9	0.37 (0.26, 0.52)
271-365 days	1	1.17 (0.74, 1.83)	8	0.67 (0.53, 0.85)	22	0.54 (0.41, 0.72)
> 365 days	1	1.00 (ref)	12	1.00 (ref)	39	1.00 (ref)

aHR: adjusted hazards-ratio; ref: reference category

[a] Frailty risk was defined using the Hospital Frailty Risk Score (HFRS), stratified into the following categories: low (HFRS<5), intermediate (HFRS 5-15) and high (HFRS>15) risk of frailty. Only results stratified by HFRS are presented for reinfected individuals; after restricting to subset of individuals who participated in the SGO survey and had CFS available, number of reinfections during Omicron was small (N=9622) and insufficient for additional analysis.

[b] Competing risks regression, controlling for age, gender, ethnicity, socioeconomic status (housing type), comorbidities, vaccination status (number of doses, time elapsed from last vaccination dose, type of vaccine [ancestral mRNA vaccine versus updated bivalent/XBB1.5 vaccine formulation), risk of frailty.

[c] COVID-19 vaccination status was determined at the point of SARS-CoV-2 infection. Full vaccination was defined as having completed a primary 2-dose vaccine series (either BNT162b2 or mRNA-1273, at least 8 weeks apart). Fully-vaccinated individuals who received additional mRNA vaccine doses at least six to nine months after the second dose were considered boosted. Additional vaccine doses were recommended to be administered around 1 year (and no earlier than 5 months) after the last dose received. Full vaccination was used as the reference category; the category of unvaccinated/partially vaccinated individuals was not presented due to the small number of unvaccinated/partially vaccinated cases in our population.

[d] Vaccination was considered effective 7 days after receipt to allow time for immune response.

Supplementary Table 7: Percentage of older adult Singaporeans with healthcare utilization prior to study period

**Percentage of older adult Singaporeans with healthcare utilization prior to study period, for purposes of calculating Hospital-Frailty-Risk Score[a]**	**Omicron cohort (N=874,160), N(%)**
**≥1 healthcare encounter (hospitalization/ED visit/outpatient visit)**	705,867 (80.74)
**≥1 hospitalization**	428,688 (49.04)
**≥2 hospitalizations**	134,863 (15.43)

ED: emergency-department

[a] Healthcare utilization was defined as any healthcare encounter (hospitalisations/emergency-department/outpatient visits) recorded in the national healthcare claims database, Mediclaims, in the preceding 4 years prior to the study period. The Mediclaims database encompasses all public/private healthcare providers in inpatient and outpatient settings; participation in the national government-administered medical-savings-scheme (Medisave) and national medical-insurance scheme (Medishield) is compulsory for Singaporeans. Frailty risk was defined using the Hospital Frailty Risk Score (HFRS), stratified into the following categories: low (HFRS<5), intermediate (HFRS 5-15) and high (HFRS>15) risk of frailty. In the minority of older adults without a healthcare encounter in the preceding 4 years, they were classified as being at low risk of frailty.

Supplementary Table 8: Risk of COVID-19 related-hospitalization and severe disease in older adult Singaporeans with ≥2 preceding hospitalisations, stratified by frailty risk, during Omicron-predominant transmission

Frailty risk	Person-years	Number of events	COVID-19 hospitalization, aHR (95%CI)[a]	Number of events	Severe COVID-19 disease, aHR (95%CI)[a]
**Omicron wave, first infections (infection-naïve)[b]**					
Low risk of frailty[c]	89224	3597	1.00 (ref)	651	1.00 (ref)
Intermediate risk of frailty[c]	56380	6901	**1.55 (1.47, 1.64)**	1525	**1.73 (1.54, 1.94)**
High risk of frailty[c]	13840	3479	**2.42 (2.28, 2.56)**	865	**2.90 (2.56, 3.29)**

aHR: adjusted hazards-ratio; ref: reference category

[a] Competing-risks-regression (Fine-Gray), taking death as a competing risk, with matching weights applied; the following variables were included in weighting: age, ethnicity, gender, socioeconomic status, comorbidity burden, and vaccination status pre-Omicron. Vaccine type (ancestral mRNA vaccine formulation versus updated vaccines [bivalent/monovalent XBB1.5 doses]), number of vaccine doses, and time-since-last-vaccination were included as covariates in the regression models.

[b] After restricting to individuals with ≥2 preceding hospitalisations for calculation of HFRS, number of reinfections during Omicron was small (N=6021) and insufficient for additional analysis.

[c] Frailty risk was defined using the Hospital Frailty Risk Score (HFRS), stratified into the following categories: low (HFRS<5), intermediate (HFRS 5-15) and high (HFRS>15) risk of frailty.

Supplementary Table 9: Risk of COVID-19 related-hospitalization and severe disease in older adult Singaporeans with ≥2 preceding hospitalisations, by number of vaccine doses and time elapsed since last vaccination, stratified by frailty risk, during Omicron-predominant transmission

	COVID-19 hospitalization						Severe COVID-19 disease					
	Low risk of frailty[a], N(%)		Intermediate risk of frailty[a], N(%)		High risk of frailty[a], N(%)		Low risk of frailty[a], N(%)		Intermediate risk of frailty[a], N(%)		High risk of frailty[a], N(%)	
	Events, per 1000 person-years	aHR (95%CI)[b]	Events, per 1000 person-years	aHR (95%CI)[b]	Events, per 1000 person-years	aHR (95%CI)[b]	Events, per 1000 person-years	aHR (95%CI)[b]	Events, per 1000 person-years	aHR (95%CI)[b]	Events, per 1000 person-years	aHR (95%CI)[b]
**Omicron wave, first infections (infection-naïve)[e]**												
**Number of vaccine doses[c]**												
2 vaccine doses	144	1.00 (ref)	296	1.00 (ref)	438	1.00 (ref)	31	1.00 (ref)	78	1.00 (ref)	127	1.00 (ref)
3 vaccine doses	41	0.56 (0.50, 0.63)	124	0.61 (0.57, 0.66)	248	0.79 (0.72, 0.87)	7	0.42 (0.33, 0.55)	25	0.45 (0.39, 0.51)	58	0.63 (0.52, 0.76)
4 vaccine doses	22	0.55 (0.45, 0.67)	68	0.69 (0.62, 0.77)	148	1.16 (1.00, 1.35)	3	0.36 (0.22, 0.57)	11	0.40 (0.31, 0.51)	29	0.91 (0.66, 1.26)
≥5 vaccine doses	11	0.53 (0.26, 1.09)	35	0.69 (0.47, 1.02)	76	1.19 (0.72, 1.99)	3	0.15 (0.03, 0.70)	4	0.27 (0.10, 0.76)	9	1.08 (0.25, 4.76)
**Time elapsed since last vaccine dose**												
0-90 days[d]	50	0.69 (0.53, 0.90)	142	0.47 (0.41, 0.54)	285	0.33 (0.28, 0.39)	10	1.51 (0.84, 2.73)	31	0.55 (0.40, 0.76)	68	0.30 (0.20, 0.45)
91-180 days	60	0.77 (0.61, 0.99)	172	0.55 (0.49, 0.63)	329	0.40 (0.34, 0.47)	10	1.45 (0.83, 2.53)	40	0.67 (0.50, 0.89)	92	0.45 (0.31, 0.66)
181-270 days	40	0.98 (0.77, 1.24)	124	0.71 (0.63, 0.80)	256	0.54 (0.46, 0.63)	9	2.25 (0.89, 3.92)	27	0.73 (0.56, 0.96)	62	0.56 (0.39, 0.80)
271-365 days	36	0.88 (0.69, 1.12)	108	0.72 (0.63, 0.81)	197	0.57 (0.48, 0.68)	6	1.43 (0.80, 2.54)	25	0.86 (0.65, 1.14)	47	0.64 (0.44, 0.93)
> 365 days	17	1.00 (ref)	61	1.00 (ref)	138	1.00 (ref)	2	1.00 (ref)	11	1.00 (ref)	26	1.00 (ref)

aHR: adjusted hazards-ratio; ref: reference category

[a] Frailty risk was defined using the Hospital Frailty Risk Score (HFRS), stratified into the following categories: low (HFRS<5), intermediate (HFRS 5-15) and high (HFRS>15) risk of frailty.

[b] Competing risks regression (Fine-Gray), controlling for age, gender, ethnicity, socioeconomic status (housing type), comorbidities, vaccination status (number of doses, time elapsed from last vaccination dose, type of vaccine [ancestral mRNA vaccine versus updated bivalent/XBB1.5 vaccine formulation), risk of frailty during Omicron-predominant transmission.

[c] COVID-19 vaccination status was determined at the point of SARS-CoV-2 infection. Full vaccination was defined as having completed a primary 2-dose vaccine series (either BNT162b2 or mRNA-1273, at least 8 weeks apart). Fully-vaccinated individuals who received additional mRNA vaccine doses at least six to nine months after the second dose were considered boosted. Additional vaccine doses were recommended to be administered around 1 year (and no earlier than 5 months) after the last dose received. Full vaccination was used as the reference category; the category of unvaccinated/partially vaccinated individuals was not presented due to the small number of unvaccinated/partially vaccinated cases in our population.

[d] Vaccination was considered effective 7 days after receipt to allow time for immune response

[e] After restricting to individuals with ≥2 preceding hospitalisations for calculation of HFRS, number of reinfections during Omicron was small (N=6021) and insufficient for additional analysis

Supplementary Table 10: Two-way cross-tabulation of frailty measures (Clinical-Frailty-Score and Hospital-Frailty-Risk-Score) in subset of the population with available data

Omicron-predominant period (N=377,944)[a]		Frailty, classified by Clinical-Frailty-Score (CFS) [b,c]			
		Moderate-severe frailty (CFS 6-8)	Mild frailty (CFS 4-5)	Non-frail (CFS 1-3)	Total
Frailty, classified by Hospital-Frailty-Risk-Score (HFRS) [b,c]	High-risk (HFRS>15)	2195 (0.6%)	1838 (0.5%)	1919 (0.5%)	5952 (1.6%)
	Intermediate-risk (HFRS 5-15)	4981 (1.3%)	9020 (2.4%)	26036 (6.9%)	40037 (10.6%)
	Low-risk (HFRS<5)	4124 (1.1%)	20137 (5.3%)	307694 (81.4%)	331955 (87.8%)
	Total	11300 (3.0%)	30995 (8.2%)	335649 (88.8%)	377944 (100.0%)
Omicron-predominant period (N=377,944)[a]		Frailty, classified by Clinical-Frailty-Score (CFS) [b,d]			
		Severe frailty (CFS 7-8)	Moderate frailty (CFS 6)	Non-frail/mild frailty (CFS 1-5)	Total
Frailty, classified by Hospital-Frailty-Risk-Score (HFRS) [b,d]	High-risk (HFRS>15)	1315 (0.3%)	880 (0.2%)	3757 (1.0%)	5952 (1.6%)
	Intermediate-risk (HFRS 5-15)	2707 (0.7%)	2274 (0.6%)	35056 (9.3%)	40037 (10.6%)
	Low-risk (HFRS<5)	2092 (0.6%)	2032 (0.5%)	327831 (86.7%)	331955 (87.8%)
	Total	6114 (1.6%)	5186 (1.4%)	366644 (97.0%)	377944 (100.0%)

[a] Study population was restricted to subset of population who participated in a large-scale nationwide survey of community-dwelling elderly, organised by the national Silver Generation Office (SGO) pre-pandemic (2019), and thus had Clinical Frailty Scale (CFS) measured as a pre-pandemic baseline, for comparison against the Hospital-Frailty-Risk-Score (HFRS), which was computed using ICD-10 diagnosis codes in national healthcare claims data, from any healthcare visit recorded in the preceding 4 years prior to start of study period.

[b] Correlation of the numerical value for HFRS and CFS was assessed using the Spearman correlation coefficient. For the Omicron cohort, the Spearman correlation coefficient for the numeric value of CFS/HFRS was 0.28.

[c] Frailty risk defined using HFRS was stratified into the following categories: low (HFRS<5), intermediate (HFRS 5-15) and high (HFRS>15) risk of frailty. Frailty risk defined using the CFS was stratified into the following categories: non-frail (CFS 1-3); mild frailty (CFS 4-5) and moderate-severe frailty (CFS 6-8). Individuals with CFS=9 were dropped as they were approaching end-of-life. The agreement between the categorised HFRS (HFRS<5, HFRS 5-15, HFRS>15) and the categorised CFS (CFS 1-3, CFS 4-5, CFS 6-8) was evaluated using weighted kappa scores; with weak agreement (weighted kappa of 0.38 for the Omicron cohort).

[d] Frailty risk defined using HFRS was stratified into the following categories: low (HFRS<5), intermediate (HFRS 5-15) and high (HFRS>15) risk of frailty. Frailty risk defined using the CFS was stratified into the following categories: non-frail/mild frailty (CFS 1-5); moderate frailty (CFS 6) and severe frailty (CFS 7-8). Individuals with CFS=9 were dropped as they were approaching end-of-life. The agreement between the categorised HFRS (HFRS<5, HFRS 5-15, HFRS>15) and the categorised CFS (CFS 1-5, CFS 6, CFS 7-8) was evaluated using weighted kappa scores; with weak agreement (weighted kappa of 0.28 for the Omicron cohort).

Supplementary Table 11: Risk of COVID-19 related-hospitalization and severe disease in older adult Singaporeans, stratified by alternative CFS grouping, during Omicron-predominant transmission

**Frailty risk**	**Number of events**	**COVID-19 hospitalization, aHR (95%CI)[a]**	**Number of events**	**Severe COVID-19 disease, aHR (95%CI)[a]**
**Omicron wave, first infections (infection-naïve)[b]**				
**Alternative CFS grouping**				
Low risk of frailty (CFS 1-3)[c]	7526	1.00 (ref)	1179	1.00 (ref)
Intermediate risk of frailty (CFS 4-5)[c]	2852	**1.44 (1.35, 1.52)**	515	**1.52 (1.32, 1.75)**
High risk of frailty (CFS 6-8)[c]	1924	**2.10 (1.97, 2.23)**	502	**2.83 (2.47, 3.24)**

aHR: adjusted hazards-ratio; ref: reference category

[a] Competing-risks-regression (Fine-Gray), taking death as a competing risk, with matching weights applied; the following variables were included in weighting: age, ethnicity, gender, socioeconomic status, comorbidity burden, and vaccination status pre-Omicron. Vaccine type (ancestral mRNA vaccine formulation versus updated vaccines [bivalent/monovalent XBB1.5 doses]), number of vaccine doses, and time-since-last-vaccination were included as covariates in the regression models.

[b] After restricting to subset of individuals who participated in the SGO survey and had CFS available, number of reinfections during Omicron was small (N=9622) and insufficient for additional analysis.

[c] Frailty risk was defined using the Clinical Frailty Scale (CFS), in subset of population who participated in a large-scale nationwide survey of community-dwelling elderly, organised by the national Silver Generation Office (SGO) pre-pandemic (2019). The CFS is rated on 9 levels: Level 1– Very Fit; Level 2 – Fit; Level 3 – Managing Well; Level 4 – Living with Very Mild Frailty; Level 5 – Living with Mild Frailty; Level 6 – Living with Moderate Frailty; Level 7 – Living with Severe Frailty; Level 8 – Living with Very Severe Frailty; Level 9 – Terminally Ill. CFS was alternatively categorised as follows: A) non-frail (CFS 1-3); mild frailty (CFS 4-5) and moderate-severe frailty (CFS 6-8). Individuals with CFS=9 were dropped as they were approaching end-of-life.

Supplementary Table 12: Risk of COVID-19 related-hospitalization and severe disease in older adult Singaporeans, by number of vaccine doses, stratified by alternative CFS grouping, during Omicron-predominant transmission

	COVID-19 hospitalization						Severe COVID-19 disease					
	Low risk of frailty (CFS 1-3)[a], N(%)		Intermediate risk of frailty (CFS 4-5)[a], N(%)		High risk of frailty (CFS 6-8)[a], N(%)		Low risk of frailty (CFS 1-3)[a], N(%)		Intermediate risk of frailty (CFS 4-5)[a], N(%)		High risk of frailty (CFS 6-8)[a], N(%)	
	Events, per 1000 person-years	aHR (95%CI)[b]	Events, per 1000 person-years	aHR (95%CI)[b]	Events, per 1000 person-years	aHR (95%CI)[b]	Events, per 1000 person-years	aHR (95%CI)[b]	Events, per 1000 person-years	aHR (95%CI)[b]	Events, per 1000 person-years	aHR (95%CI)[b]
**Omicron wave, first infections (infection-naïve)[d]**												
**Number of vaccine doses[c]**												
2 vaccine doses	73	1.00 (ref)	176	1.00 (ref)	234	1.00 (ref)	16	1.00 (ref)	38	1.00 (ref)	72	1.00 (ref)
3 vaccine doses	17	0.49 (0.44, 0.55)	67	0.56 (0.51, 0.63)	138	0.82 (0.72, 0.93)	2	0.38 (0.30, 0.49)	11	0.42 (0.33, 0.53)	33	0.59 (0.47, 0.75)
4 vaccine doses	10	0.47 (0.39, 0.55)	42	0.57 (0.48, 0.67)	80	0.99 (0.81, 1.20)	1	0.29 (0.19, 0.43)	6	0.33 (0.22, 0.50)	14	0.56 (0.37, 1.01)
≥5 vaccine doses	6	0.44 (0.24, 0.80)	23	0.39 (0.22, 0.70)	59	1.13 (0.59, 2.14)	1	0.14 (0.03, 0.64)	2	0.25 (0.05, 1.26)	11	0.78 (0.18, 3.32)

aHR: adjusted hazards-ratio; ref: reference category

[a] Frailty risk was defined using the Clinical Frailty Scale (CFS), in subset of population who participated in a large-scale nationwide survey of community-dwelling elderly, organised by the national Silver Generation Office (SGO) pre-pandemic (2019). The CFS is rated on 9 levels: Level 1– Very Fit; Level 2 – Fit; Level 3 – Managing Well; Level 4 – Living with Very Mild Frailty; Level 5 – Living with Mild Frailty; Level 6 – Living with Moderate Frailty; Level 7 – Living with Severe Frailty; Level 8 – Living with Very Severe Frailty; Level 9 – Terminally Ill. CFS was categorised as follows: non-frail (CFS 1-3); mild frailty (CFS 4-5) and moderate-severe frailty (CFS 6-8). Individuals with CFS=9 were dropped as they were approaching end-of-life.

[b] Competing risks regression (Fine-Gray), controlling for age, gender, ethnicity, socioeconomic status (housing type), comorbidities, vaccination status (number of doses, time elapsed from last vaccination dose, type of vaccine (ancestral mRNA vaccine versus updated bivalent/XBB1.5 vaccine formulation), risk of frailty during Omicron-predominant transmission.

[c] COVID-19 vaccination status was determined at the point of SARS-CoV-2 infection. Full vaccination was defined as having completed a primary 2-dose vaccine series (either BNT162b2 or mRNA-1273, at least 8 weeks apart). Fully-vaccinated individuals who received additional mRNA vaccine doses at least six to nine months after the second dose were considered boosted. Additional vaccine doses were recommended to be administered around 1 year (and no earlier than 5 months) after the last dose received. Full vaccination was used as the reference category; the category of unvaccinated/partially vaccinated individuals was not presented due to the small number of unvaccinated/partially vaccinated cases in our population. Vaccination was considered effective 7 days after receipt to allow time for immune response.

[d] After restricting to subset of individuals who participated in the SGO survey and had CFS available, number of reinfections during Omicron was small (N=9622) and insufficient for additional analysis.

Supplementary Figure 1: Cohort construction flowchart for subset of population with baseline Clinical Frailty Scale (CFS) data

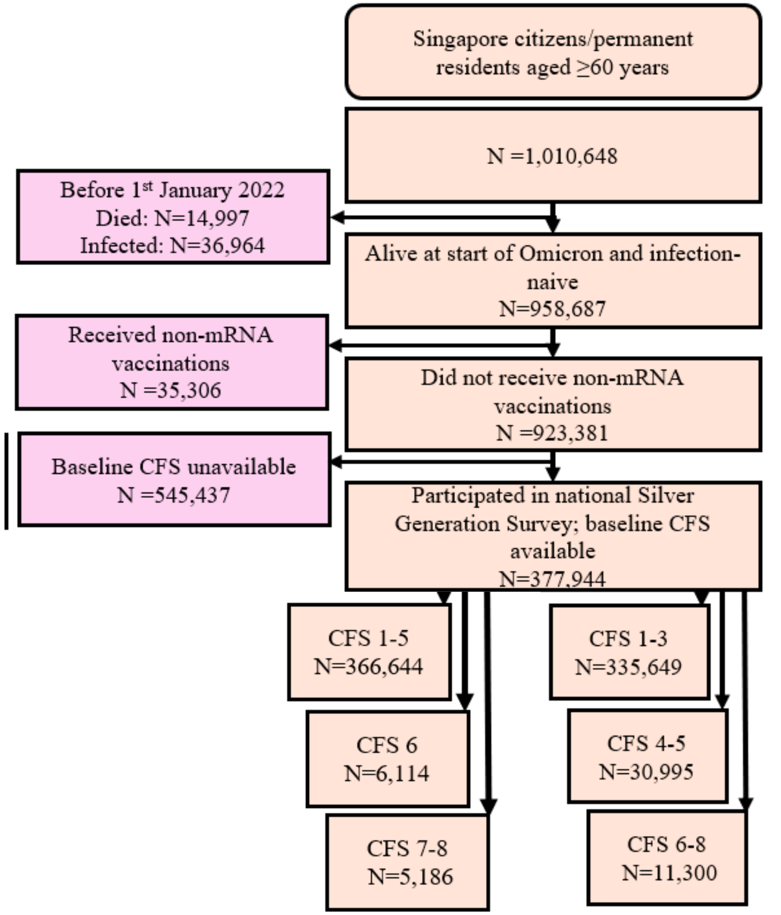

Frailty risk was defined using the Clinical Frailty Scale (CFS), in subset of population who participated at baseline in a large-scale nationwide survey of community-dwelling elderly, organised by the national Silver Generation Office (SGO) pre-pandemic (2019). Individuals with CFS=9 (N=227) were dropped as they were approaching end-of-life.
